# Psychometric properties of the Arabic version of the confusion assessment method for the intensive care unit (CAM-ICU)

**DOI:** 10.1186/s12888-018-1676-0

**Published:** 2018-04-06

**Authors:** Maha H. Aljuaid, Ahmad M. Deeb, Maamoun Dbsawy, Daniah Alsayegh, Moteb Alotaibi, Yaseen M. Arabi

**Affiliations:** 1 King Abdullah International Medical Research Center, Nursing Department, King Abdulaziz Medical City, Ministry of National Guard Health Affairs, King Saud bin Abdulaziz University for Health Sciences, Riyadh, Saudi Arabia; 2 King Abdullah International Medical Research Center, Research Office, King Abdulaziz Medical City, Ministry of National Guard Health Affairs, King Saud bin Abdulaziz University for Health Sciences, Riyadh, Saudi Arabia; 3 King Abdullah International Medical Research Center, Intensive Care Department, King Abdulaziz Medical City, Ministry of National Guard Health Affairs, King Saud bin Abdulaziz University for Health Sciences, Riyadh, Saudi Arabia; 4 King Abdullah International Medical Research Center, Mental Health Department, King Abdulaziz Medical City, Ministry of National Guard Health Affairs, King Saud bin Abdulaziz University for Health Sciences, Riyadh, Saudi Arabia; 5 King Abdullah International Medical Research Center, College of Medicine, King Saud Bin Abdulaziz University for Health Sciences, Riyadh, Saudi Arabia; 6 Intensive Care Department, King Abdulaziz Medical City, National Guard Health Affairs, Riyadh, Saudi Arabia

**Keywords:** Delirium, CAM-ICU, Arabic, Critical care, Mechanical ventilation, Geriatric, Cognition, Inattention

## Abstract

**Background:**

It is recommended that critically ill patients undergo routine delirium monitoring with a valid and reliable tool such as the Confusion Assessment Method for the Intensive Care Unit (CAM-ICU). However, the validity and reliability of the Arabic version of the CAM-ICU has not been investigated. Here, we test the validity and reliability of the Arabic CAM-ICU.

**Methods:**

We conducted a psychometric study at ICUs in a tertiary-care hospital in Saudi Arabia. We recruited consecutive adult Arabic-speaking patients, who had stayed in the ICU for at least 24 hours, and had a Richmond Agitation-Sedation Scale (RASS) score ≥ − 2 at examination. Two well-trained examiners (ICU nurse and intensivist) independently assessed delirium in eligible patients with the Arabic CAM-ICU. Evaluations by the two examiners were compared with psychiatrist blind clinical assessment of delirium according to the Diagnostic and Statistical Manual of Mental Disorders, Fifth Edition (DSM-5). Subgroup analyses were conducted for age, invasive mechanical ventilation, and gender.

**Results:**

We included 108 patients (mean age: 62.6 ± 17.6; male: 51.9%), of whom 37% were on invasive mechanical ventilation. Delirium was diagnosed in 63% of enrolled patients as per the psychiatrist clinical assessment. The Arabic CAM-ICU sensitivity was 74% (95% confidence interval [CI] = 0.63–0.84) and 56% (95%CI = 0.44–0.68) for the ICU nurse and intensivist, respectively. Specificity was 98% (95%CI = 0.93–1.0) and 92% (95%CI = 0.84–1.0), respectively. Sensitivity was greater for mechanically-ventilated patients, women, and those aged ≥65 years. Specificity was greater for those aged < 65 years, non-mechanically-ventilated patients and men. The median duration to complete the Arabic CAM-ICU was 2 min (interquartile range, 2–3) and 4.5 min (IQR, 3–5) for the ICU nurse and intensivist, respectively. Inter-rater reliability (kappa) was 0.66.

**Conclusions:**

The Arabic CAM-ICU demonstrated acceptable reliability and validity to assess delirium in Arabic-speaking ICU patients.

## Background

Delirium is defined as a disturbance in attention and awareness that fluctuates over a short period of time and accompanied by changes in cognition or perceptual disturbances [1]. Delirium is extremely common in critically ill patients, resulting from the acute illness, comorbidities, and use of sedation and analgesia. Furthermore, delirium is influenced by environmental and physical factors in intensive care unit (ICU) settings, such as isolation, limited visiting hours, the use of restraint, and connection to multiple tubes, lines, and catheters [2–5].

Previous studies report that delirium occurs in between 21% and 84% of critically ill patients [6–11]. Moreover, delirium is linked to poor ICU and hospital outcomes, such as increased morbidity, increased length of stay at the ICU and hospital, self-extubation, removal of lines and catheters, and increased treatment cost [5, 10–14]. In addition, delirium is associated with increased morbidity and mortality, [11, 15, 16] and increased cognitive and functional deficits after hospital discharge [17, 18].

Routine monitoring of delirium is considered as a quality indicator for optimum care in older patients [19], and was recommended in 2013 by clinical practice guidelines for the management of pain, agitation, and delirium in adult patients in the ICU [20]. However, delirium is underdiagnosed and monitoring of delirium tends to be sporadic and underperformed [13, 21–23]. Therefore, the routine use of valid and reliable tools to assess for the presence of delirium is recommended at least once per ICU shift for patients with a high risk of delirium [20].

The Confusion Assessment Method for the ICU (CAM-ICU) is a rapid, reliable, and valid tool to assess delirium in the ICU [6]. The CAM-ICU is an adapted version of the bedside assessment tool for non-psychiatrists, the Confusion Assessment Method (CAM), which was based on the Diagnostic and Statistical Manual of Mental Disorders, Third Edition, Revised (DSM-III-R) [24]. The CAM-ICU demonstrates good validity and reliability when administered by ICU nurses and physicians for both ventilated and non-ventilated ICU patients [6, 25–27].

The CAM-ICU has been translated into several languages, with high reliability and validity [28–33]. Indeed, the CAM-ICU has been translated into Arabic by the ICU Delirium and Cognitive Impairment Study Group at Vanderbilt University Medical Center for Health Services Research [34]. However, the tool has not yet been tested for validity and reliability. The use of a valid and reliable Arabic delirium assessment tool is crucial to diagnose, prevent, and manage delirium among Arabic-speaking ICU patients.

In this study, we assess the psychometric properties of the current Arabic version of the CAM-ICU and its applicability to Arabic-speaking critical care patients’ at a tertiary care hospital in Saudi Arabia.

## Methods

### Setting

We conducted this psychometric validation study at the Intensive Care Department (ICD) in King Abdulaziz Medical City (KAMC), Riyadh, Saudi Arabia. KAMC is a 1000-bed teaching tertiary care centre accredited by the Joint Commission International (JCI). The ICD encompasses a 21-bed medical ICU, 9-bed surgical ICU, 8-bed trauma ICU, 8-bed neuro-critical care unit, and 14-bed intermediate care unit. These units are staffed by onsite board-certified intensivists for 24 hours a day, 7 days a week. The average nurse: patient ratio is 1:1. Clinical rounds are multidisciplinary and include physicians, critical care nurses, clinical pharmacists, dieticians, physiotherapists and respiratory therapists. This study was approved by the institutional review board of KAMC and funded by the King Abdullah International Medical Research Center.

All study participants or substitute decision-makers (for patients lacking decision-making capacity) signed informed consent before participation. A consecutive sample technique was used to obtain the targeted sample size. Screening was conducted twice weekly during the day shift in the ICD between April 2016 and March 2017.

### Study participants

In this study, we included Arabic-speaking critically ill patients at the ICD, aged ≥18 years and with a Richmond Agitation-Sedation Scale (RASS) ≥ − 2, who had stayed for at least 24 hours in the ICU. We excluded patients with the following: pre-existing severe dementia or psychosis; neurologic diseases including traumatic brain injury, acute brain lesion, or spinal cord injury; burns; patients admitted because of drug overdose; patients with blindness or deafness; those with Glasgow Coma Scale < 9 at the time of screening; patients unable to complete assessments as they are medically unstable; and patients/substitute decision-makers who refused to sign informed consent form and participate in the study.

### Sample size

Previous studies have reported that delirium occurs in between 21% and 84% of critically ill patients [6–11]. Based on previous non-English CAM-ICU validation studies, sensitivity ranges from 73% to 92% and specificity ranges from 72% to 100% [29–31, 33, 35]. Accordingly, we expected values for sensitivity and specificity of 85%. Therefore, with an estimated mid-percentage of delirium prevalence of 50%, and an acceptable width of 95% confidence interval (CI) for sensitivity and specificity to no greater than 10% (75–95%), we calculated a minimum required sample size of 98 [36]. To account for potential dropouts and to compensate for assessments that could not be done within 4 hours by all assessors or missing assessment by any assessor, we anticipated a sample size of 125 patients.

### Procedures

The CAM-ICU has already been translated into Arabic by the ICU Delirium and Cognitive Impairment Study Group at Vanderbilt University Medical Center for Health Services Research. The tool (both visual and auditory sections) was translated to suit the cultural and educational background of Arabic-speaking populations. The translations were conducted independently by two members before they discussed it together. The final Arabic version was then back translated to the original language (English) by a professional independent translator [34].

The Arabic CAM-ICU comprises the same four features that are used in the English CAM-ICU to screen patients for delirium. These features include: 1) acute onset of mental status changes or a fluctuating course; 2) inattention; and 3) altered level of consciousness; or 4) disorganized thinking. For inattention, the Arabic CAM-ICU uses mainly number sequences (e.g. 6 3 1 1 4 1 5 7 1 8) instead of letter sequences (e.g. S A V E A H A A R T). However, using alternate sequences of Arabic letters is possible. In addition, some words were changed in items assessing disorganized thinking to suit the Arabic speaking culture (e.g. pounds changed to kilos) [34]. As with the CAM-ICU, a patient is considered delirious if he/she test positive for features 1 and 2, in addition to being positive for either feature 3 or 4 [34]. The Arabic CAM-ICU and the training manual are available from the ICU Delirium and Cognitive Impairment Study Group website [34].

In our validation study, two well-trained examiners (an ICU Clinical Nurse Specialist and Intensivist) independently assessed delirium in eligible ICU patients with the Arabic CAM-ICU. They performed the CAM-ICU in the same group of patients but without any specific order, with an allowed duration of up to 4 hours between two exams for each patient. All assessments were performed during the day shift.

In addition, an experienced psychiatrist specialising in geriatric psychiatry independently assessed the same ICU patients for delirium according to the Diagnostic and Statistical Manual of Mental Disorders, Fifth Edition (DSM-5) criteria [1] within the same 4 hours window of evaluation as the ICU examiners. All assessors were blinded to the results of the others until the end of the study. The results of the psychiatric screening were compared with those of the ICU examiners screening. All assessors are authors in this study.

#### Data collection

The following data was recorded: date and time of assessments; assessment location; gender; age; use of mechanical ventilation; RASS score [37]; and delirium assessment results (Arabic CAM-ICU and psychiatrist clinical assessment).

#### Statistical analysis

The psychometric properties of the Arabic CAM-ICU were calculated for the ICU nurse and intensivist separately compared with the psychiatrist clinical assessment of delirium. We calculated the sensitivity, specificity, positive likelihood ratio (LR+), negative likelihood ratio (LR-), positive predictive values, negative predictive values, pretest probability, pretest odds, posttest odds, posttest probability, accuracies, and prevalence. Subgroup analyses were conducted for age, invasive mechanical ventilation status, and gender.

Agreement (inter-rater reliability) was assessed by calculating Cohen’s kappa. Continuous data are represented by mean ± standard deviation (SD) or median (quartile Q1, Q2). Calculations were performed with the Statistical Package for the Social Sciences version 24 (SPSS Inc., Chicago, IL, USA).

## Results

### Patient characteristics

From 129 patients, a total of 125 patients agreed to participate and were enrolled in the study. Of these, 17 patients were excluded from the study analysis for the following reasons: duration of assessments between examiners exceeded the allowed 4 hours (*n* = 13); diagnosis of chronic schizophrenia (*n* = 1); diagnosis with drug addiction (*n* = 1); psychiatrist clinical assessment of delirium not completed (*n* = 1); and psychiatrist clinical assessment of delirium missing (*n* = 1).

We included 108 patients in the final analysis, among which the CAM-ICU was conducted by a nurse and the clinical assessment of delirium by psychiatrist in 108 patients, and the CAM-ICU was conducted by an intensivist for 106 patients. Approximately half of the patients were male (51.9%), and the mean age of participants was 62.6 ± 17.6 years with a median of 66 years (interquartile range: 63.8–75 years). The majority of participants were not on invasive mechanical ventilation (*n* = 68, 63%).

With the psychiatrist clinical assessment, delirium was diagnosed in 68 patients (63%).

### Psychometric properties of the Arabic CAM-ICU conducted by an ICU nurse

The Arabic CAM-ICU conducted by an ICU nurse identified 51 delirious patients (47.2%). We calculated a sensitivity of 74% (95%CI = 63–84%) and a specificity of 98% (95%CI = 93–100%). The LR+ was 29.4 (95%CI = 29.4–29.5) and the LR- was 0.27 (95%CI = 0.15–0.39). Table 1 presents the other psychometric properties of the Arabic CAM-ICU conducted by an ICU nurse. The median duration to complete the Arabic CAM-ICU by the nurse was 2 min (interquartile range: 2–3 min).

**Table 1:** Psychometric properties of the Arabic CAM-ICU

Property	CAM-ICU by nurse (*n* = 108)	CAM-ICU by intensivist (*n* = 106)
Sensitivity (95%CI)	0.74 (0.63–0.84)	0.56 (0.44–0.68)
Specificity (95%CI)	0.98 (0.93–1.0)	0.92 (0.84–1.0)
Likelihood ratio, LR+ (95%CI)	29.4 (29.4–29.5)	7.1 (7–7.2)
Likelihood ratio, LR- (95%CI)	0.27 (0.15–0.39)	0.48 (0.36–0.60)
Positive predicted value (95%CI)	0.98 (0.94–1.0)	0.93 (0.85–1.0)
Negative predicted value (95%CI)	0.68 (0.56–0.81)	0.54 (0.42–0.66)
Pre-test probability	0.63	0.64
Pre-test odds	1.7	1.79
Post-test odds	50	12.7
Post-test probability	0.98	0.93
Delirium prevalence, CAM-ICU vs. psychiatrist clinical assessment	47.2% vs. 63%	38.7% vs. 64.2%
Accuracy	82.4%	68.9%

*CAM-ICU* Confusion Assessment Method for the Intensive Care Unit, *CI* Confidence interval

We conducted subgroup analyses for age group, mechanical ventilation status, and gender. In patients older than or equal 65 years, the sensitivity was 79% (95%CI = 67–91%) and the specificity was 95% (95%CI = 85–100%). In patients younger than 65 years, the sensitivity was 64% (95%CI = 45–83%) and the specificity was 100% (95%CI = 100–100%). Table 2 presents other psychometric properties of the Arabic CAM-ICU according to age.

**Table 2:** Psychometric properties of the Arabic CAM-ICU according to age

Property	CAM-ICU by nurse (*n* = 62)	CAM-ICU by intensivist (*n* = 62)
Age ≥ 65 years		
Sensitivity (95%CI)	0.79 (0.67–0.91)	0.65 (0.51–0.79)
Specificity (95%CI)	0.95 (0.85–1.0)	0.90 (0.76–1.0)
Likelihood Ratio LR+ (95%CI)	15.0 (15.0–15.1)	6.2 (6.1–6.3)
Likelihood Ratio LR- (95%CI)	0.22 (0.04–0.40)	0.39 (0.22–0.56)
Positive predicted value (95%CI)	0.97 (0.92–1.0)	0.93 (0.84–1.0)
Negative predicted value (95%CI)	0.67 (0.49–0.84)	0.53 (0.36–0.70)
Pre-test probability	0.69	0.69
Pre-test odds	2.26	2.26
Post-test odds	34.00	14
Post-test probability	0.97	0.93
Delirium prevalence, CAM-ICU vs. psychiatrist clinical assessment	56.5% vs. 69.4%	48.4% vs. 69.4%
Accuracy	83.9%	72.6%
Age < 65 years		
Sensitivity (95%CI)	0.64 (0.45–0.83)	0.40 (0.21–0.59)
Specificity (95%CI)	1.0 (1.0–1.0)	0.95 (0.85–1.0)
Likelihood Ratio LR+ (95%CI)	Undefined*	7.6 (7.4–7.8)
Likelihood Ratio LR- (95%CI)	0.36 (0.20–0.52)	0.63 (0.46–0.80)
Positive predicted value (95%CI)	1.0 (1.0–1.0)	0.91 (0.74–1.1)
Negative predicted value (95%CI)	0.70 (0.54–0.86)	0.55 (0.38–0.72)
Pre-test probability	0.54	0.57
Pre-test odds	1.19	1.32
Post-test odds	Undefined**	10
Post-test probability	Undefined***	0.91
Delirium prevalence, CAM-ICU vs. psychiatrist clinical assessment	34.8% vs. 54.4%	25% vs. 56.8%
Accuracy	80.4%	63.6%

*CAM-ICU* Confusion Assessment Method for the Intensive Care Unit, *CI* confidence interval *Specificity value is 1; **Likelihood Ratio is undefined; ***Post-test odd is undefined

In patients with invasive mechanical ventilation, the sensitivity was 75% (95%CI = 58–92%) and the specificity was 94% (95%CI = 82–100%). In patients without invasive mechanical ventilation, the sensitivity was 73% (95%CI = 60–86%) and the specificity was 100% (95%100–100%). Table 3 presents other psychometric properties of the Arabic CAM-ICU according to invasive mechanical ventilation status.

**Table 3:** Psychometric properties of the Arabic CAM-ICU according to mechanical ventilation status

Property	CAM-ICU by nurse (*n* = 40)	CAM-ICU by intensivist (*n* = 39)
Mechanical ventilation		
Sensitivity (95%CI)	0.75 (0.58–0.92)	0.70 (0.51–0.88)
Specificity (95%CI)	0.94 (0.82–1.0)	0.88 (0.71–1.0)
Likelihood Ratio LR+ (95%CI)	12 (11.9–12.1)	5.57 (5.4–5.7)
Likelihood Ratio LR- (95%CI)	0.27 (0.07–0.46)	0.35 (0.15–0.55)
Positive predicted value (95%CI)	0.95 (0.85–1.0)	0.89 (0.74–1.0)
Negative predicted value (95%CI)	0.71 (0.52–0.91)	0.67 (0.47–0.87)
Pre-test probability	0.60	0.59
Pre-test odds	1.5	1.44
Post-test odds	18.0	8.0
Post-test probability	0.95	0.89
Delirium prevalence, CAM-ICU vs. psychiatrist clinical assessment	47.5% vs. 60%	46.2% vs. 59%
Accuracy	82.5%	76.9%
No mechanical ventilation		
Sensitivity (95%CI)	0.73 (0.60–0.86)	0.50 (0.34–0.64)
Specificity (95%CI)	1.0 (1.0–1.0)	0.96 (0.87–1.0)
Likelihood Ratio LR+ (95%CI)	Undefined*	10.8 (10.7–10.8)
Likelihood Ratio LR- (95%CI)	0.27 (0.12–0.43)	0.54 (0.39–0.68)
Positive predicted value (95%CI)	1.0 (1.0–1.0)	0.96 (0.87–1.0)
Negative predicted value (95%CI)	0.67 (0.51–0.82)	0.48 (0.33–0.63)
Pre-test probability	0.65	0.67
Pre-test odds	1.83	2.05
Post-test odds	Undefined**	22.0
Post-test probability	Undefined***	0.96
Delirium prevalence, CAM-ICU vs. psychiatrist clinical assessment	47.1% vs. 64.7%	34.3% vs. 67.2%
Accuracy	82.4%	64.2%

*CAM-ICU* Confusion Assessment Method for the Intensive Care Unit, *CI* confidence interval * Specificity value is 1; ** Likelihood Ratio is undefined; *** Post-test odd is undefined

In male patients, the sensitivity was 71% (95%CI = 55–86%) and the specificity was 100% (95%CI = 100–100%). In female patients, the sensitivity was 77% (95%CI = 62–91%) and the specificity was 94% (95%CI = 84–100%). Table 4 presents other psychometric properties of the Arabic CAM-ICU according to gender.

**Table 4:** Psychometric properties of the Arabic CAM-ICU according to gender

Property	CAM-ICU by nurse (*n* = 56)	CAM-ICU by intensivist (*n* = 54)
Male		
Sensitivity (95%CI)	0.71 (0.55–0.86)	0.47 (0.30–0.64)
Specificity (95%CI)	1.0 (1.0–1.0)	0.95 (0.85–1.0)
Likelihood Ratio LR+ (95%CI)	Undefined*	9.4 (9.3–9.5)
Likelihood Ratio LR- (95%CI)	0.29 (0.13–0.46)	0.56 (0.40–0.72)
Positive predicted value (95%CI)	1.0 (1.0–1.0)	0.94 (0.83–1.0)
Negative predicted value (95%CI)	0.69 (0.53–0.85)	0.51 (0.35–0.67)
Pre-test probability	0.61	0.63
Pre-test odds	1.55	1.70
Post-test odds	Undefined**	16
Post-test probability	Undefined***	0.94
Delirium prevalence, CAM-ICU vs. psychiatrist clinical assessment	42.9% vs. 60.7%	31.5% vs. 63%
Accuracy	82.1%	64.8%
Female		
Sensitivity (95%CI)	0.77 (0.62–0.91)	0.65 (0.49–0.81)
Specificity (95%CI)	0.94 (0.84–1.0)	0.89 (0.74–1.0)
Likelihood Ratio LR+ (95%CI)	13.8 (13.7–13.8)	5.82 (5.71–5.93)
Likelihood Ratio LR- (95%CI)	0.25 (0.07–0.43)	0.40 (0.21–0.58)
Positive predicted value (95%CI)	0.96 (0.89–1.0)	0.92 (0.81–1.0)
Negative predicted value (95%CI)	0.68 (0.50–0.86)	0.57 (0.39–0.76)
Pre-test probability	0.65	0.65
Pre-test odds	1.89	1.89
Post-test odds	26.0	11
Post-test probability	0.96	0.92
Delirium prevalence, CAM-ICU vs. psychiatrist clinical assessment	51.9% vs. 65.4%	46.2% vs. 65.4%
Accuracy	82.7%	73.1%

*CAM-ICU* Confusion Assessment Method for the Intensive Care Unit, *CI* confidence interval * Specificity value is 1; ** Likelihood Ratio is undefined; *** Post-test odd is undefined

### Psychometric properties of the Arabic CAM-ICU conducted by an intensivist

The Arabic CAM-ICU conducted by an intensivist identified 41 delirious patients (38.7%), with a sensitivity of 56% (95%CI = 44–68%), and a specificity of 92% (95%CI = 84–100%). The LR+ was 7.1 (95%CI = 7–7.2) and the LR- was 0.48 (95%CI = 0.36–0.60). Table 1 presents other psychometric properties of the Arabic CAM-ICU conducted by an intensivist. The median duration to complete the Arabic CAM-ICU by an intensivist was 4.5 min (interquartile range: 3–5 min).

We conducted subgroup analyses for age group, mechanical ventilation status, and gender. In patients older than or equal 65 years, the sensitivity was 65% (95%CI = 51–79%) and the specificity was 90% (95%CI = 76–100%). In patients younger than 65 years, the sensitivity was 40% (95%CI = 21–59%) and the specificity was 95% (95%CI = 85–100%). Table 2 presents other psychometric properties of the Arabic CAM-ICU according to age.

In patients with invasive mechanical ventilation, the sensitivity was 70% (95%CI = 51–88%) and the specificity was 88% (95%CI = 71–100%). In patients without invasive mechanical ventilation, the sensitivity was 50% (95%CI = 34–64%) and the specificity was 96% (95%CI = 87–100%). Table 3 presents other psychometric properties of the Arabic CAM-ICU according to invasive mechanical ventilation status.

In male patients, the sensitivity was 47% (95%CI = 30–64%) and the specificity was 95% (95%CI = 85–100%). In female patients, the sensitivity was 65% (95%CI = 49–81%) and the specificity was 89% (95%CI = 74–100%). Table 4 presents other psychometric properties of the Arabic CAM-ICU according to gender.

### Reliability of the Arabic CAM-ICU

We assessed the level of agreement between assessments with the Arabic CAM-ICU conducted by the ICU nurse and intensivist by calculating inter-rater reliability (kappa). Assessments were matched in 88 patients (83%) with a kappa of 0.66 (standard error: 0.07; *p* < 0.0001).

## Discussion

In this study, we demonstrated the psychometric properties of the Arabic CAM-ICU. In this study, patients were assessed for delirium by an intensivist and ICU nurse with the Arabic CAM-ICU, and these assessments were compared with the psychiatrist clinical assessment according to DSM-5. In addition, subgroup analyses were conducted for age, mechanical ventilation status, and gender to determine the validity of the Arabic CAM-ICU in specific groups.

Previous studies have reported that delirium occurs in between 21% and 84% of critically ill patients [6–11]. In this study, the psychiatrist clinical assessment identified delirium in 63% of critically ill patients. This data suggests that delirium is similarly common among critically ill patients in the Arabic-speaking population. This high rate of delirium highlights the importance of a reliable and valid measure to assess delirium for critically ill patients in the ICU. In addition, it suggests the need for urgent action to establish delirium treatment and prevention measures.

Assessment of the Arabic CAM-ICU by the ICU nurse had a sensitivity of 74%. The sensitivity increased to 75% for mechanically ventilated patients, 77% for female patients, and 79% for patients aged ≥65 years. Although sensitivity in our study is less than that reported in the initial validation study for the English CAM-ICU by Ely et al. (93–100%) [6, 27], it was within the ranges reported by other validation studies for non-English versions of the CAM-ICU (73–92%) [29–33, 35]. Therefore, we believe that the sensitivity for the Arabic CAM-ICU is acceptable.

Assessment of the Arabic CAM-ICU by the ICU nurse had a specificity of 98%. The specificity increased to 100% for patients aged < 65 years, non-mechanically ventilated patients, and male patients. The specificity of the Arabic CAM-ICU is consistent with that reported by Ely et al. (89–100%) [6, 27], which suggests that the specificity of the Arabic CAM-ICU is acceptable. Taking our results together, the Arabic CAM-ICU appears to be a valid tool for the assessment of delirium among Arabic-speaking critically ill patients.

Nonetheless, our results did show some discrepancies between raters. Although comparable values for specificity were achieved between raters (98% vs. 92%), there was greater discrepancy in sensitivity values (74% vs. 56%). These discrepancies have not been reported in other studies that have used nurses and intensivists as CAM-ICU raters [27, 32]. In the study by Ely et al., sensitivity values of the CAM-ICU for two nurses and an intensivist were 95%, 96%, and 100%, respectively, and specificity values were 93%, 93%, and 89%, respectively [27]. Moreover, in a Spanish study, sensitivity values of the CAM-ICU for an ICU nurse and an intensivist were 83% and 80%, respectively, and specificity was 96% for both raters [32]. Nonetheless, in our study, the raters’ assessments were matched in 83% patients with a kappa of 0.66, which is considered moderate and acceptable inter-rater reliability [38]. We believe that the accuracy of the CAM-ICU assessment could be further improved by providing proper education about delirium and training for the CAM-ICU [33, 39, 40].

This is the first study to assess the reliability and validity of the Arabic CAM-ICU as a delirium assessment tool for critically ill patients. Our study has several strengths including a relatively large and powered sample size. In addition, it measured inclusive psychometric features of the Arabic CAM-ICU. Furthermore, we conducted subgroup analyses to measure the psychometric features of the Arabic CAM-ICU in specific groups. This is the first study to highlight delirium rate for Arabic-speaking critically ill patients. The main limitation of the study is that it assessed inattention components of the Arabic CAM-ICU with the auditory attention screening exam only. We recommend that for future studies to investigate the reliability and validity of the visual attention screening exam of the Arabic CAM-ICU. In addition, we excluded patients with neurological diseases including traumatic brain injury; such neurological diseases may dispose patients to different neuropsychiatric consequences [41]. The CAM-ICU may not be the optimal tool to assess delirium in such patients [42]. Developing specific tools to assess and differentiate delirium from other neuropsychiatric dysfunctions is recommended for future research [42].

## Conclusions

The Arabic CAM-ICU demonstrated acceptable reliability and validity to assess delirium in Arabic-speaking ICU patients. We recommend researchers and clinicians to use the Arabic CAM-ICU as delirium assessment tool for Arabic-speaking ICU patients, taking into consideration the knowledge and training needed of delirium assessment. Further studies including the Arabic CAM-ICU would identify its further strengths and limitations in research and clinical fields.

## Abbreviations

CAM: Confusion Assessment Method
CAM-ICU: Confusion assessment method for the intensive care unit
CI: Confidence interval
DSM-5: Diagnostic and Statistical Manual of Mental Disorders, Fifth Edition
ICU: Intensive care unit
IQR: Interquartile range
JCI: Joint Commission International
KAMC: King Abdulaziz Medical City
LR +: Positive likelihood ratio
LR-: Negative likelihood ratio
RASS: Richmond Agitation-Sedation Scale
SD: Standard deviations
